# A 45-year-old man with sudden cardiac death, cutaneous abnormalities and a rare desmoplakin mutation: a case report and literature review

**DOI:** 10.1186/s12872-022-02472-5

**Published:** 2022-02-12

**Authors:** Cátia Santos-Ferreira, Rui Baptista, Tiago Teixeira, Lino Gonçalves

**Affiliations:** 1 Cardiology Department, Centro Hospitalar e Universitário de Coimbra, Praceta Professor Mota Pinto, 3000-075 Coimbra, Portugal; 2 Cardiology Department, Centro Hospitalar Entre Douro e Vouga, Santa Maria de Feira, Portugal; 3 Coimbra Institute for Clinical and Biomedical Research (iCBR), Faculty of Medicine, Univ Coimbra, Coimbra, Portugal

**Keywords:** Arrhythmogenic cardiomyopathy, Dilated cardiomyopathy, Sudden cardiac death, Desmoplakin mutation, Cutaneous abnormalities, Case report

## Abstract

**Background:**

Arrhythmogenic cardiomyopathy (AC) is a rare, heritable myocardial disorder that is a leading cause of ventricular arrhythmia and sudden cardiac death (SCD) in young people. Desmoplakin (*DSP*) mutations account for 3–20% of AC cases. However, the number of patients with *DSP* mutations is extremely small in all published reports and genotype–phenotype correlations are scant and mostly non-gene-specific.

**Case presentation:**

A 45-year-old man was admitted after an out-of-hospital cardiac arrest, with documented ventricular fibrillation. He had no previous history of heart disease or family history of SCD or cardiomyopathy. The cardiac magnetic resonance showed a mildly dilated left ventricle with an ejection fraction of 30% and a non-dilated right ventricle with mildly depressed systolic function, and extensive subepicardial late gadolinium enhancement. Genetic screening identified a heterozygote nonsense mutation in *DSP* (NM_004415.2: c.478 C > T; p.Arg160Ter). Cascade genetic screening of the relatives revealed a high prevalence of the genotype and cutaneous phenotype, but a very low penetrance of the cardiac phenotype.

**Conclusions:**

We report a case of SCD and an autosomal dominant mutation in *DSP* that causes arrhythmogenic dilated cardiomyopathy/AC. Like the recessive mutation in *DSP* known to cause Carvajal syndrome, Arg160Ter may be associated with cutaneous abnormalities.

## Background

Arrhythmogenic cardiomyopathy (AC) is a rare, heritable myocardial disorder that is a leading cause of ventricular arrhythmia and sudden cardiac death (SCD) in people age ≤ 35 years [1, 2]. Progressive loss of myocardium and its replacement by fibrofatty tissue is the pathological hallmark of the disease [2]. AC is most well recognized in its classic subtype with right-sided preponderance, arrhythmogenic right ventricular cardiomyopathy [3]. More recently, a broader spectrum of disorders affecting either or both ventricles and an increased propensity to ventricular arrhythmias has been recognized, arousing many questions regarding pathogenesis and clinical management of AC [4, 5].

There is no single gold standard for the diagnosis, which is mainly based on demonstrating characteristic electrical, structural, and/or histological abnormalities. In addition, a positive family history for a pathogenic genetic mutation also contributes to the diagnosis [6]. Mutations in the genes encoding proteins of the desmosomal complex account for 30 to 70% of cases, namely plakoglobin (*JUP),* plakophilin-2 (*PKP2*)*,* desmoplakin *(DSP),* desmocollin-2 (*DSC2)*, and desmoglein-2 (*DSG2)* [1, 7]. Noteworthy, pathogenic desmosomal mutations are also common in patients with dilated cardiomyopathy (DCM), including 3% of cases in a recent large DCM cohort [8]. Inheritance of AC is classically considered autosomal dominant with age-related, reduced penetrance, and variable expressivity [9]. Autosomal recessive forms are rare but recognized, most prominently in the cardiocutaneous syndromes of Naxos and Carvajal [1, 10, 11]. Nonetheless, emerging evidence suggests that many cases of AC are oligogenic or even multifactorial with both genomic and environmental factors contributing to pathogenesis [12]. *DSP* mutations account for 3–20% of AC cases [1, 9, 12, 13]. ﻿However, the number of patients with *DSP* mutations is extremely small in all published reports and genotype–phenotype correlations are scant and mostly non-gene-specific [13].

Here, we describe a novel heterozygote nonsense mutation c.478C > T (p.Arg160Ter) in the gene encoding *DSP* leading to DCM/AC presenting as SCD.

## Case presentation

**Fig. 1:**
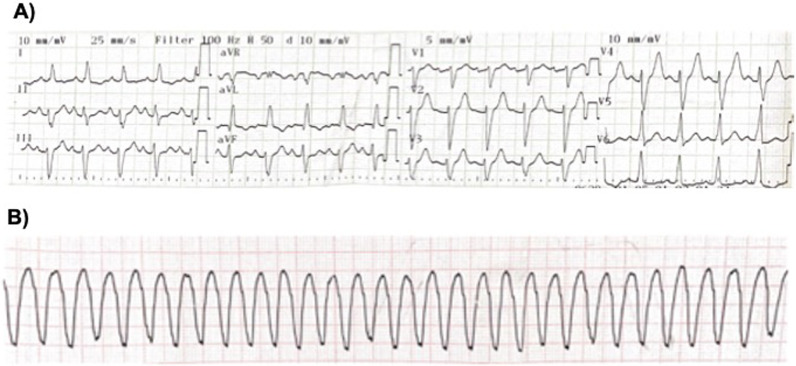
**A** Twelve-lead electrocardiogram after aborted cardiac arrest. **B** Rhythm strip showing monomorphic ventricular tachycardia

**Fig. 2:**
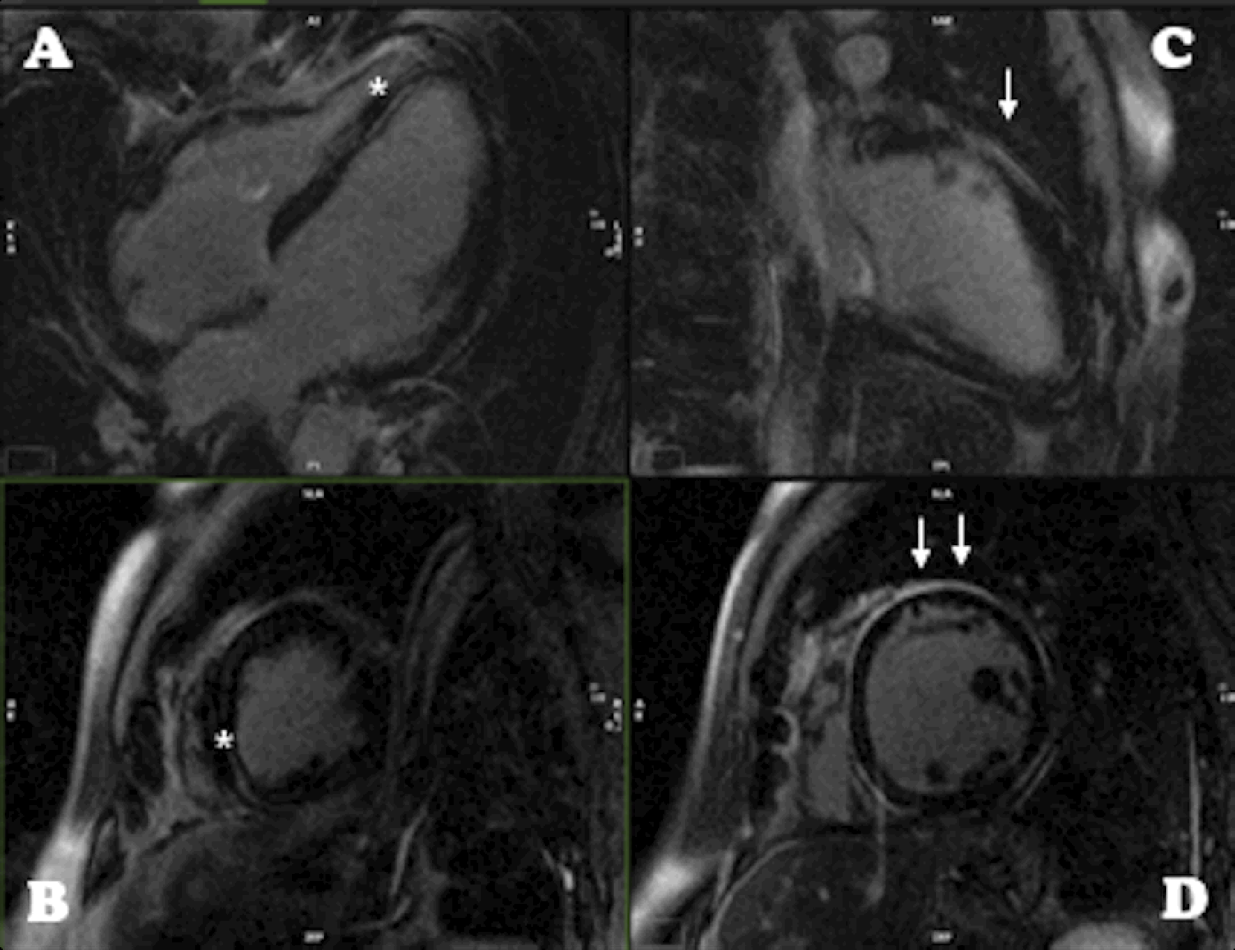
Cardiac magnetic resonance late gadolinium enhancement images showing typical pattern: intramyocardial (*) linear lesion in (**A**) and (**B**), long and correspondent short-axis respectively, here affecting the inferior septum; very extensive subepicardial scar (arrow) in (**C**) and (**D**), again long and correspondent short-axis respectively, here affecting particularly the anterior and anterolateral wall, but being almost circumferential and also affecting the right apex (**A**)

A 45-year-old man was admitted after an out-of-hospital cardiac arrest, with documented ventricular fibrillation. He had no viral prodrome, previous history of heart disease, or family history of SCD or cardiomyopathy. Besides interventricular conduction delay, the baseline electrocardiogram showed T wave inversion in the lateral leads (Fig. 1A). Myocardial ischemia due to epicardial obstructive coronary artery disease was excluded by coronary angiography. The echocardiogram performed on admission revealed a dilated left ventricle (LV) with severely depressed LV ejection fraction (LVEF of 29%); a right ventricle (RV) with preserved function; and cardiac valves and pericardium were normal. The patient evolved into cardiogenic shock and vasopressor and inotropic support was started with norepinephrine and dobutamine. During the first 48 hours of hospitalization, the patient presented one episode of sustained monomorphic ventricular tachycardia with right bundle branch block (RBBB) morphology requiring electrical cardioversion (Fig. 1B). The cardiac magnetic resonance (CMR) showed a mildly dilated LV with an LVEF of 30% due to global hypokinesis; a non-dilated RV with mildly depressed systolic function; and an extensive subepicardial pattern of late gadolinium enhancement (LGE-CMR), almost circumferential in some regions, along with disperse intramyocardial lesions (Fig. 2). No mediastinal lymphadenopathy or pulmonary granulomas were seen. After an implantable cardioverter-defibrillator implantation, the patient was discharged under an angiotensin receptor–neprilysin inhibitor, beta-blocker, mineralocorticoid antagonist, and antiarrhythmic treatment with amiodarone.

**Fig. 3:**
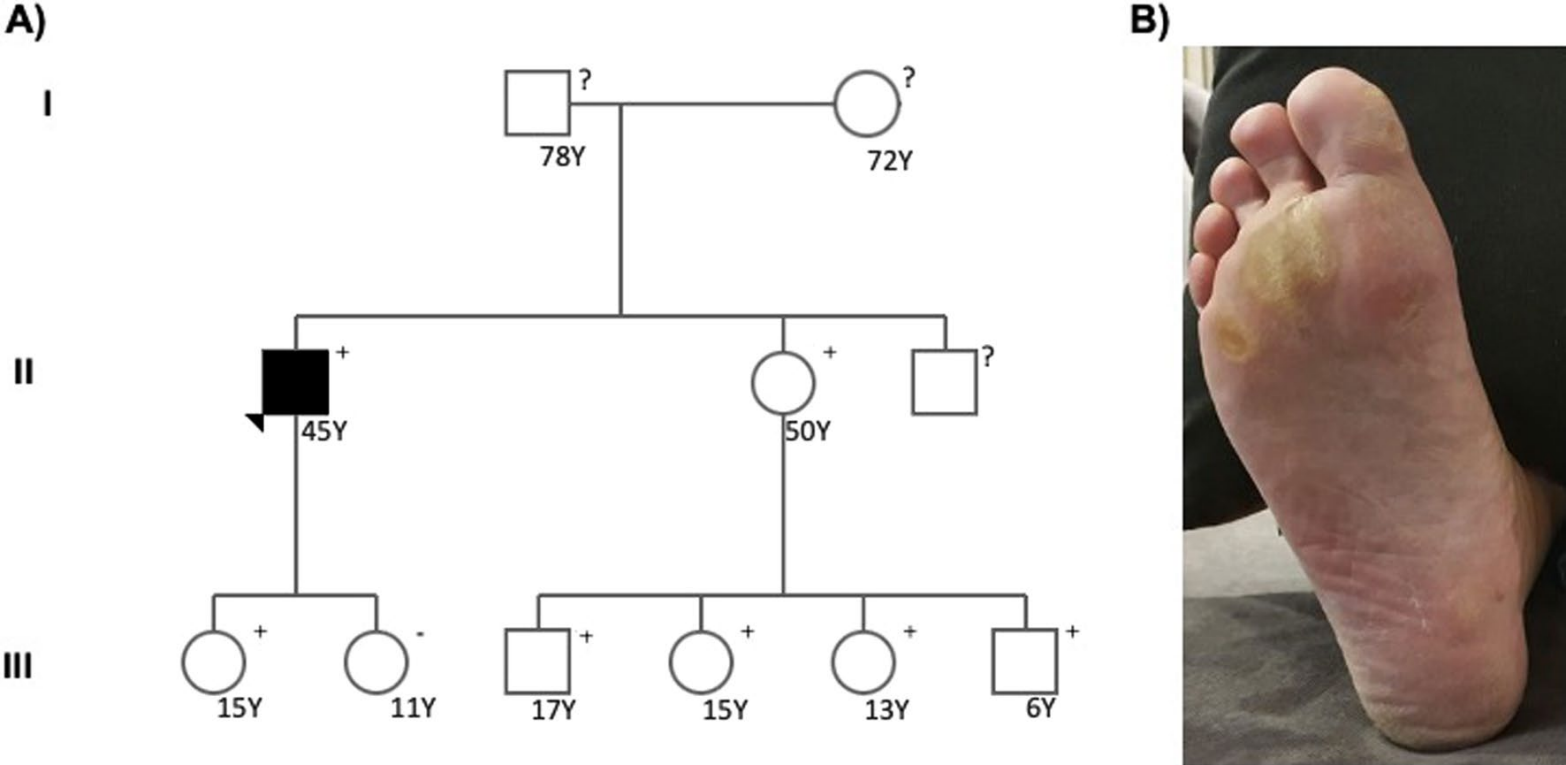
**A** Pedigree of the family. The index case is indicated by an arrow. Cases with the p.Arg160Ter in the DSP gene mutation are shown as (+), and those without the mutation are shown as (−). Squares represent males, and circles represent females. Black-filled symbols represent individuals with cardiac phenotype or symptoms. **B** Palmoplantar keratoderma affecting index patient’s sister

Analysis of the index patient’s genomic DNA revealed a nonsense mutation in *DSP* (NM_004415.2: c.478C > T; p.Arg160Ter). Cascade genetic screening of the relatives revealed a high prevalence of the genotype and cutaneous phenotype (curly hair and palmoplantar keratoderma), but a very low penetrance of the cardiac phenotype (only the index case) (Fig. 3).

During the next 24 months of follow-up, neither symptoms nor implantable cardioverter-defibrillator therapy recurred and a LVEF recovery to 45–50% was observed.

## Discussion and conclusions

Genetic testing is widely recommended in SCD when there is a cardiac phenotype, including only genes with robust gene-disease association [14]. Nonetheless, growing evidence supports a broad, multi-phenotype genetic testing even without a suspected phenotype, as there is the notion that pathogenic variants in cardiomyopathy genes may result in SCD without overt structural changes [15–17].

The combination of aborted cardiac death/ventricular tachycardia with RBBB morphology and a CMR with biventricular dysfunction and a non-ischemic pattern of LGE-CMR raised the suspicion of sarcoidosis, myocarditis, AC, and DCM. Undeniably, genetic testing was crucial for the final diagnosis.

**Fig. 4:**
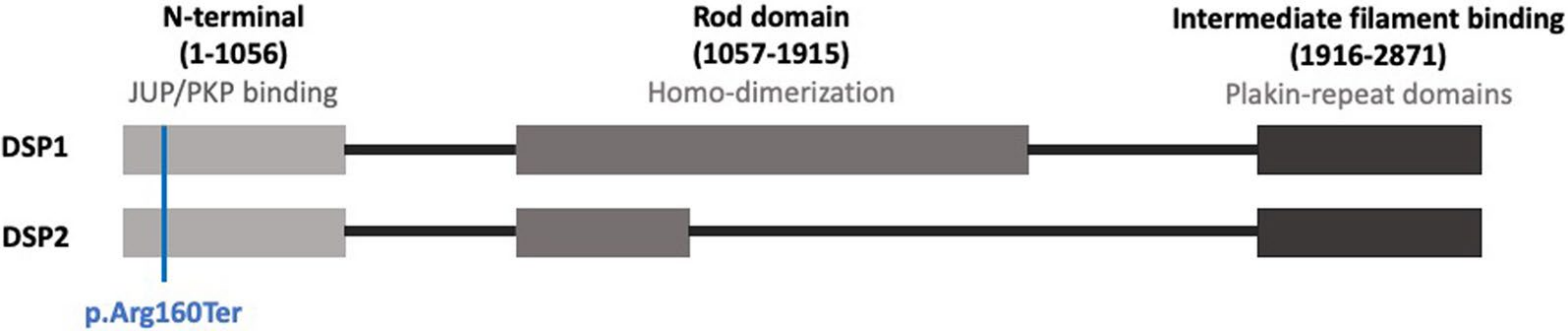
Structure of the DSP gene and the Arg160Ter mutation

We identified a nonsense mutation in the *DSP* gene (c.478C > T; p.Arg160Ter), which was absent from large population studies. This nonsense variant leads to a premature termination codon at position 160, which is predicted to lead to a truncated protein. The DSP is a large protein (2871 AA) composed of three domains characterized by an N-terminal plakoglobin/plakophilin binding domain, the central coiled-coil rod dimerization domain, and a C-terminal intermediate filament binding domain [18]. The *DSP* gene encodes two different splice isoforms produced by alternate splicing (*DSP1* and *DSP2*). The longer peptide DSP-1 is the dominant isoform in heart [19], whereas DSP-2 uses an internal splice donor site and therefore has two-thirds fewer amino acids within the central domain compared to DSP-1 [20]. The Arg160Ter mutation is located in the first domain and hence appears in both isoforms (Fig. 4). This pathogenic variant in the *DSP* gene has been reported several times in association with AC and DCM [21].

*DSP* is an essential component of the desmosome in epithelial cells and cardiomyocytes. Several human *DSP* variants have been linked to inherited diseases that variably result in skin fragility, palmoplantar keratoderma, woolly hair, and cardiomyopathies [22]. Nevertheless, a clear phenotype-genotype correlation regarding *DSP* mutations has not yet been drawn.

There are many diagnostic challenges associated with AC, particularly with the predominant LV involvement (ALVC) form of the disease, that may not display the classic revised 2010 Task Force Criteria and exhibits considerable genetic and phenotypic overlap with DCM [23, 24]. More recently, the “Padua criteria” for the diagnosis of AC included specific criteria for ALVC [25]. Nevertheless, in the absence of RV involvement that meets arrhythmogenic right ventricular cardiomyopathy criteria, the diagnosis relies heavily on a pathogenic mutation combined with CMR results [25]. The distinction between the AC and DCM in our patient was difficult. First, the mutation identified in DSP has been described in both cardiomyopathies [21]. Second, the ECG failed to show low voltage in limb leads, a low specificity but characteristic pattern of ALVC [23]. Additionally, while it revealed T wave inversion in lateral leads which may be present in ALVC, the interventricular conduction delay is more frequently present in DCM [24]. Third, the clinical presentation of SCD in a previously asymptomatic patient favors ALVC diagnosis, as well as the monomorphic sustained ventricular tachycardia with RBBB morphology, the latter is a minor criterion for ALVC [24, 25]. Lastly, although our patient presented severe systolic dysfunction, he had only mild LV dilatation and a large amount of non-ischemic LGE-CMR, a finding more typical in ALVC, as impairment of the LV is often present without scar in DCM [24, 26]. Remarkably, the morpho-functional and structural LV abnormalities are minor and major criteria for ALVC diagnosis, respectively [25].

AC is characterized by incomplete and age-dependent penetrance coupled with variable phenotypic expression [1]. *DSP* mutations are associated with more penetrant phenotypes with an increased arrhythmic propensity, which is often the first manifestation of the disease [13], as was the case with our patient. Secondly, DSP mutations seem to be more often associated with a predominant LV phenotype or biventricular involvement [13]. Lastly, although autosomal dominant AC forms have been reported as cardiac-specific, a cutaneous phenotype has been described in DSP mutation carriers [22, 27]. A highly penetrant cutaneous phenotype of curly hair and to a lesser extent, palmoplantar keratoderma, is particularly associated with dominant, nonsense, or frameshift mutations falling to the N-terminal domain of DSP [27], just like the mutation described in this family, that besides curly hair also presented the palmoplantar features (Fig. 3B). Increased awareness of this phenotype may facilitate a timely diagnosis of AC in the absence of overt cardiac features [27].

Importantly, cascade screening revealed a high prevalence of the genotype and cutaneous phenotype, but a very low penetrance of the cardiac phenotype. Indeed, the index patient was the only family member with overt cardiac disease. Remarkably, he was the only member of the family who had practiced high-intensity exercise, which is in agreement with the concept that the volume of exercise has a critical influence on the evolution of AC [12].

Nevertheless, the crucial question is how to predict the risk of a major arrhythmic event among asymptomatic mutation carriers [28], as SCD may be the form of presentation. The most important factors to consider when determining arrhythmic risk in AC include: (1) electrical instability, (2) proband status, (3) manifest structural disease, (4) cardiac syncope, (5) male gender, (6) multiple mutations or a mutation in TMEM 43, and (7) vigorous exercise [29].

The arrhythmic risk in AC-causing desmosomal mutation carriers significantly increases when the AC phenotype becomes overt [30, 31]. The major challenge is the detection of structural disease in ALVC, particularly in *DSP*-mutation carriers [30]. The so-called ‘concealed phase’ of the disease may be a result of the low sensitivity of routine clinical tests, namely ECG and echocardiography [28]. The value of LGE-CMR to detect fibrosis as an early finding of LV involvement in still asymptomatic and not fulfilling criteria for AC diagnosis is emerging [5]. The most distinctive feature of ALVC phenotype is the large amount of LV myocardial fibrosis, which is directly related to the LV systolic dysfunction [24, 26], and is ﻿characteristically in the posterolateral and septal LV wall [32–34]. More recently, a subepicardial ring-like scar pattern was found to be characteristic in the DSP genotype and is likely to have diagnostic value in ALVC [26]. Interestingly, our index patient showed such a typical pattern, which allowed suspicion of AC. The non-invasive early LV tissue characterization highlights the LGE-CMR potential in broadening the diagnostic criteria for AC and improving risk stratification [6, 32, 35]. However, further studies are warranted to assess whether and when systematic evaluation of DSP-mutation carriers with LGE-CMR in addition to traditional tests may improve our ability to stratify the arrhythmic risk in AC mutation carriers [26, 28, 30].

This case highlights the importance of genetic testing in the determination of arrhythmic cardiac arrest etiology. Here, we present an autosomal dominant mutation in *DSP* that causes arrhythmogenic DCM/AC. Like the recessive mutation in *DSP* known to cause Carvajal syndrome, Arg160Ter may be associated with cutaneous abnormalities. Further studies are needed to improve our understanding of AC, and ultimately, provide a more accurate diagnostic algorithm and improved genetics counseling and management strategies.

## Data Availability

Not applicable.

## Abbreviations

AC: Arrhythmogenic cardiomyopathy
ALVC: Arrhythmogenic cardiomyopathy with predominant LV involvement
CMR: Cardiac magnetic resonance
DCM: Dilated cardiomyopathy
DSP: Desmoplakin
LGE-CMR: Late gadolinium enhancement—cardiac magnetic resonance
LV: Left ventricle
LVEF: Left ventricular ejection fraction
RBBB: Right bundle branch block
RV: Right ventricle
SCD: Sudden cardiac death
